# An Agar-Based Method for Plating Marine Protozoan Parasites of the Genus *Perkinsus*

**DOI:** 10.1371/journal.pone.0155015

**Published:** 2016-05-05

**Authors:** Emma R. Cold, Nastasia J. Freyria, Joaquín Martínez Martínez, José A. Fernández Robledo

**Affiliations:** 1 Bigelow Laboratory for Ocean Sciences, East Boothbay, Maine, United States of America; 2 Research Experiences for Undergraduates (REU) NSF Program - 2015 - Bigelow Laboratory for Ocean Sciences, Boothbay, Maine, United States of America; 3 Université de Toulon, Toulon, France; University of Camerino, ITALY

## Abstract

The genus *Perkinsus* includes protozoan parasites of mollusks responsible for losses in the aquaculture industry and hampering the recovery of natural shellfish beds worldwide, and they are a key taxon for understanding intracellular parasitism adaptations. The ability to propagate the parasite in liquid media, in the absence of the host, has been crucial for improving understanding of its biology; however, alternative techniques to grow the parasite are needed to explore other basic aspects of the *Perkinsus* spp. biology. We optimized a DME: Ham’s F12–5% FBS- containing solid agar medium for plating *Perkinsus marinus*. This solid medium supported trophozoite propagation both by binary fission and schizogony. Colonies were visible to the naked eye 17 days after plating. We tested the suitability of this method for several applications, including the following: 1) Subcloning *P*. *marinus* isolates: single discrete *P*. *marinus* colonies were obtained from DME: Ham’s F12–5% FBS– 0.75% agar plates, which could be further propagated in liquid medium; 2) Subcloning engineered *Perkinsus mediterraneus* MOE[MOE]: GFP by streaking cultures on plates; 3) Chemical susceptibility: Infusing the DME: Ham’s F12–5% FBS– 0.75% agar plates with triclosan resulted in inhibition of the parasite propagation in a dose-dependent manner. Altogether, our plating method has the potential for becoming a key tool for investigating diverse aspects of *Perkinsus* spp. biology, developing new molecular tools, and for biotechnological applications.

## Introduction

Protozoan parasites significantly affect wild and farmed mollusk species around the world (OIE; http://www.oie.int/; Aquatic Animal Health Code, Section 11: Diseases of Mollusks). Most protozoan parasites have complex life cycles with most of the life cycle stages being intracellular; consequently, culture of the parasite requires the culture of either host cell lines or primary cells. *Perkinsus* spp. are the only protozoan parasites of mollusks that can be grown *in vitro* in the absence of the host cells [1–3]. Arguably (the affiliation *Perkinsus qugwadi* is uncertain [4]), the genus *Perkinsus* includes six species with five of them in culture and available at a public repository (American Type Culture Collection, USA, [5]). The ease of culturing *Perkinsus* spp. has prompted many studies and publications addressing diverse aspects of the genus *Perkinsus*' biology [6] and has allowed the generation of numerous tools and resources including the sequencing of *P*. *marinus*’ genome [7], transcriptomic and proteomic profiles [8–11], and the development of a transfection system [12]. This transfection system has been used to unravel subcellular mechanisms crucial to *Perkinsus* spp. survival inside oyster hemocytes [13]. Additionally, it is a key tool to genetically engineering *P*. *marinus* to induce systemic immunity against infectious agents and to produce recombinant proteins of medical and veterinary interest [14, 15]. Gene regulation in the genus *Perkinsus* is by transplacing, a process that converts a polycistronic transcript into monocistronic mRNAs by incorporating a 22-bp RNA fragment (splice leader) into the 5**’** end of independently transcribed pre-mRNAs to yield mature mRNAs [16, 17]. This particular way of regulating gene expression has limited the development of transfection vectors, which, in the absence of clear gene promoters, relies on using gene-flanking regions [12]. So far, no resistance cassette for positive selection has been developed for the *P*. *marinus* transfection system with identification of the transfectants relying on tagging genes with fluorescence tags (*e*.*g*. green fluorescent protein, GFP) and subcloning the transfectants by selecting fluorescent cells by limiting dilution or manually pipetting individual fluorescent cells [12]. The ability to grow cells onto solid media plates can facilitate subcloning and may become crucial for selecting *Perkinsus* spp. transfectants once specific resistance cassettes become available. The ability to propagate *Perkinsus* spp. in the absence of host cells makes them appropriate candidates for cultivation onto solid media plates, although such methods have not been developed yet.

In addition to subcloning, major applications of plating include chemosensitivity testing, strain phenotyping based on colony morphology, tropism analysis, extracellular product secretion analysis, and mutagenesis, among others [18–24]. In this study, we developed a method for plating *P*. *marinus* in Dulbecco’s modified Eagle medium (DME): Ham’s F12–5% FBS solidified with agar. We also engineered *Perkinsus mediterraneus* for expressing GFP and the fluorescent cells were cloned using plating. We further investigated the applicability of our plating technique to study the effect of drugs on *P*. *marinus*. As a proof of concept, we tested infusing the solid media with triclosan, a known *Perkinsus* spp. inhibitor. The plating methodology is straightforward and it can be easily implemented; we also discuss other the potential applications of the plating methodology.

## Materials and Methods

### Parasite strains and *in vitro* culture

Cultures of the wild-type *P*. *marinus* ATCC PRA-240 and *P*. *mediterraneus* ATCC PRA-238 [25] were maintained in DME: Ham's F12 (1:2) supplemented with 5% fetal bovine serum (FBS) in 25 cm^2^ (5–8 ml) polystyrene canted neck cell culture flasks with vent caps (Corning^®^, Corning, NY) at 26–28°C in a microbiology incubator as reported elsewhere [26].

### Plate preparation, *P*. *marinus* plating, and subcloning

Equal volumes of double-strength sterile bacteriological agar (Sigma-Aldrich, St. Louis, MO) and double-strength liquid DME: Ham’s F-12 medium containing 10% FBS, were mixed with both solutions at 52°C. The mixture was immediately poured (15 ml or -5–7 ml) into Petri dishes (100 mm x 15 mm or 60 mm x 15 mm) (VWR, Radnor, PA) and allowed to set at room temperature under sterile conditions. Plates could then be stored at 4°C until being used. Solid media plates at final agar concentrations of 0.65, 0.75, 1.25, and 1.5% were prepared for testing. These agar concentrations had been previously tested for cultivation of other protozoan parasite [19]. Prior to plating, a *P*. *marinus* culture in log phase was diluted in culture medium to 2,000 cells ml^-1^, and 0.5 ml were evenly spread by rotation onto the different agar concentration-media plates in triplicate. Inocula were allowed to adsorb for 15 min before moving plates to the 26–28°C incubator. The plates were monitored over time by eye and under an inverted microscope (Olympus IX70, Center Valley, PA) and colonies and lawn formation were photographically documented (Olympus TG-3; Canon EOS Rebel T3 18.0 MP SLR, Melville, NY). *P*. *marinus* colonies were subcloned by excising them from the plate using a sterile 1 ml pipette tip with the tip cut off, and depositing them in 3.0 ml liquid culture medium in wells of 6-well plates. The plates were incubated under the same conditions indicated above.

### *Perkinsus mediterraneus* transfection and subcloning

*Perkinsus mediterraneus* trophozoites in the log phase were resuspended in 100 μl of Amaxa’s solutions V and electroporated with 5 μg *p*PmMOE-GFP-11 (2.5 μg supercoiled, 2.5 μg *Not*I linearized) using the D-023 program in a Nucleofector^™^ II (Lonza, Walkersville, MD) [12]. Transfected cultures were monitored over time for green fluorescence using standard FITC excitation/ emission filters (488/507 nm) under an Olympus IX-70 transmitted-light fluorescence microscope. For subcloning transfectants expressing GFP, cells from a culture containing both transfected and non-transfected cells were plated by spreading 100 μl of culture onto the solid media and by streaking the plates using a microbiology loop. GFP-transfected colonies, i.e. with green fluorescence, were picked and streaked again on plates for sub-cloning. Three colonies were then picked from the plate using a sterile pipette tip and deposited in 0.7 ml of fresh medium in a 48-well plate for culture expansion and cryopreservation at -80°C. The monoclonal culture of *P*. *mediterraneus* MOE[MOE]:GFP-B5 was deposited in the ATCC (accession number pending).

### Chemical infused agar plating

Once the optimal concentration of agar was determined (0.75%), we prepared a set of plates containing triclosan [5-chloro-2-(2.4 dichlorophenoxy)-phenol] (Sigma-Aldrich) at different concentrations. The stock solution was prepared in 100% ethanol and diluted in the DME: Ham’s F12–5% FBS (2x) prior to mixing with agar medium to yield final concentrations of 50, 100, and 200 μM; final concentration of ethanol on the plates was 1% or less, a concentration known to have no negative effects on *Perkinsus* spp. viability [27, 28]. The prepared plates were allowed to set and were kept at room temperature for 24 hours for sterility checking. Biological replicates (n = 9) with 2 ×10^5^ *P*. *marinus* trophozoites in 5 μl of medium were spotted on the plates containing 0.75% of agar, incubated at 28°C, and monitored daily for up to 21 days. Controls included *P*. *marinus* plated onto plates with no triclosan.

## Results and Discussion

### Plating and subcloning of plated colonies

**Fig 1:**
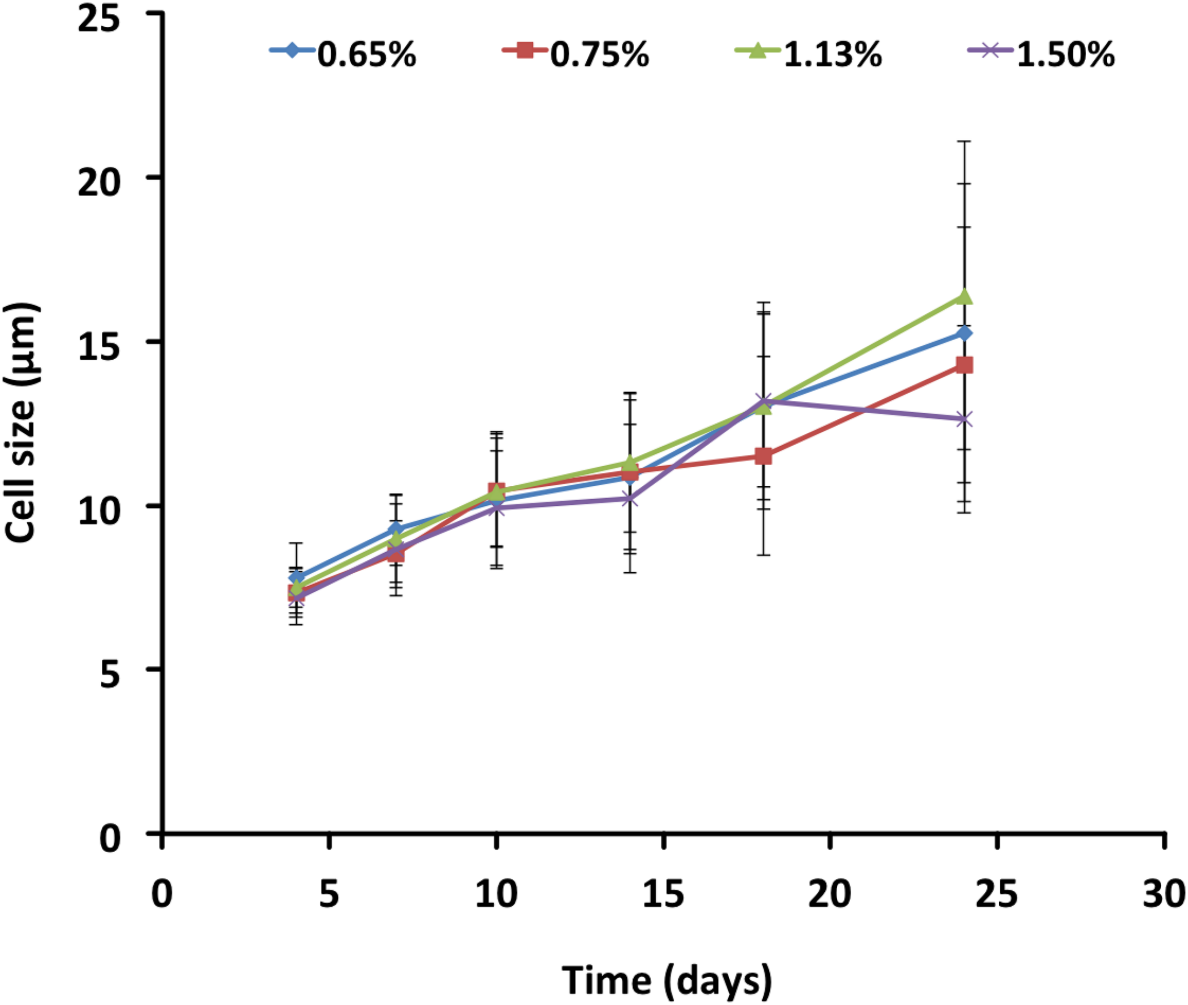
*Perkinsus marinus* trophozoites grown on agar plates. Cell size of *P*. *marinus* trophozoites plated on plates containing variable percentage of agar.

**Fig 2:**
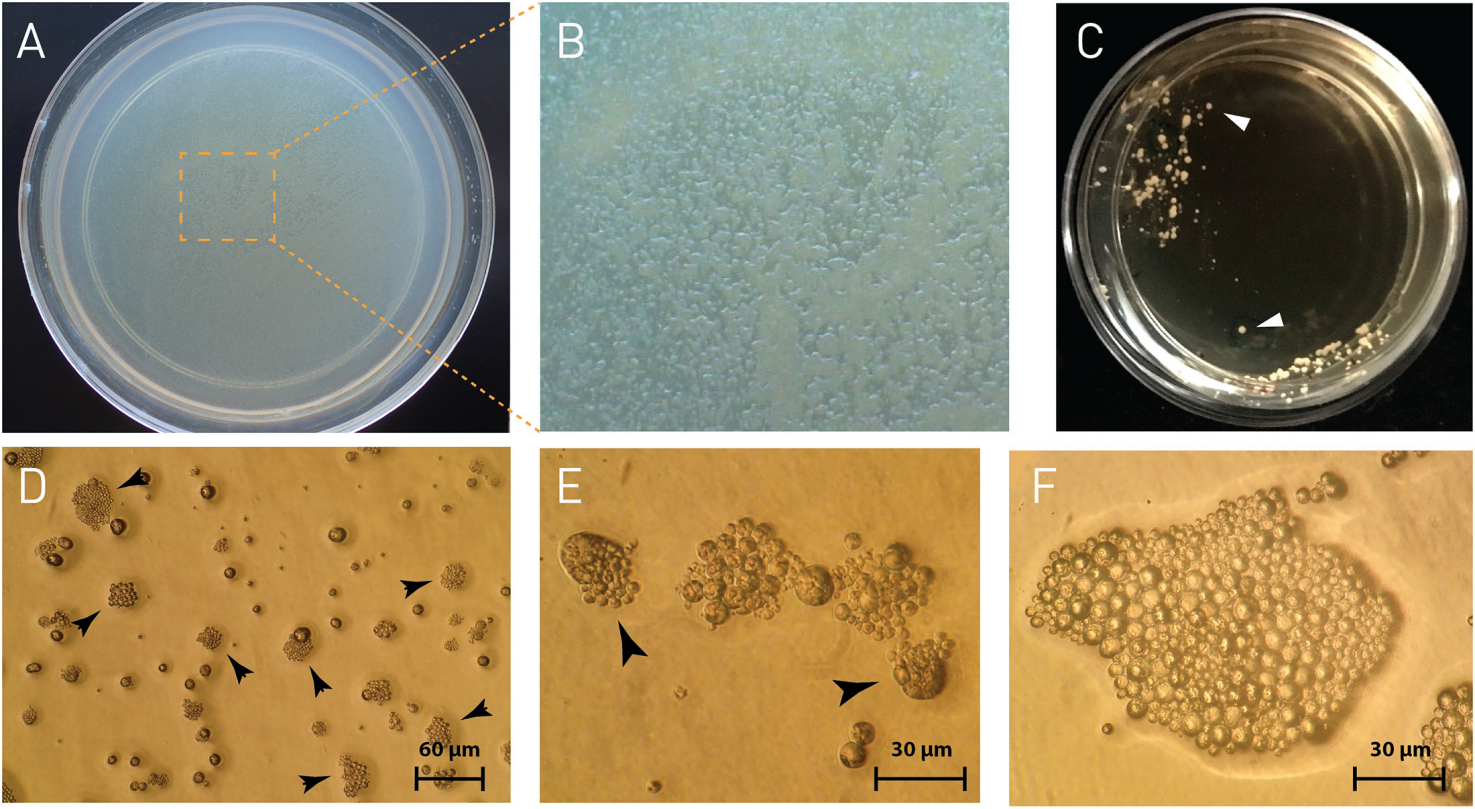
*Perkinsus* spp. colonies on agar plates. **A.** General appearance of *P*. *marinus* colonies and lawn growing on agar plates (60 mm x 15 mm). **B.** Close up of square on **A** showing colonies and *P*. *marinus* lawn. **C.** General appearance of *P*. *mediterraneus* colonies after streaking the cultures on agar plates, note individual colonies (arrowheads). **D.** Low magnification of *P*. *marinus* colonies (arrowheads). **E.** Detail of *P*. *marinus* schizogony; note the daughter cells being released from the mother cell (arrowheads). **F.** Large *P*. *marinus* colony; note that cell division is not synchronized as indicated by the differences in cell size.

The suitability of the solid media was assessed primarily by whether or not *P*. *marinus* was able to propagate. We also considered the ease of manipulating the plates and the colonies based on the medium consistency and the transparency of the medium to be able to see the colonies under the inverted microscope. Plates of all agar concentrations resulted in the formation of *P*. *marinus* colonies and lawn and no differences in cell size were observed (Fig 1). However, 0.75% agar medium was employed for subsequent experiments, as it was easier to pour (even spreading in the plates) and its consistency once solidified allowed for easy manipulation and observation of the colonies under an inverted microscope. Additionally, thinner medium layers of 4–6 mm enhanced microscopic observations and photo documentation. Discrete colonies were obtained by distributing the liquid medium containing *P*. *marinus* on the entire surface using clock- and anticlockwise rotation (Fig 2A and 2B) or by streaking the plate with an inoculating loop (Fig 2C). It took at least 17 days for *P*. *marinus* colonies to appear visible to the naked eye; this can be explained by *P*. *marinus*’ relatively slow doubling time (17–24 hours) [1, 26]. Although, *P*. *marinus* colonies require more time than bacteria/yeast to be visible to the naked eye on the plates, the agar-based medium can support *P*. *marinus* growth for extended periods (we have exceeded six-month-old plates with *P*. *marinus*, results not shown). Trophozoites propagate mostly by schizogony (asexual reproduction by multiple fission of the parasite’s nucleus followed by cytoplasmic segmentation), while binary fission and budding are seldom observed [29]. Indeed, in plated *P*. *marinus*, schizogony was the main propagation strategy observed (Fig 2D and 2E); though we also observed trophozoites propagating by binary fission and uneven groups of three and five cells indicating non-synchronic division. In *Trypanosoma brucei* it has been suggested that agar mimics an interaction of trypanosomes with the host's extracellular matrix and direct physical contact with the agar matrix is essential for maintaining the ability to differentiate from replicating long slender bloodstream forms into short stumpy forms (pleomorphic infections) as happens during infection of mice [22]. In this study we saw no effect of the agar concentration on the trophozoite size over a period of three weeks.

### Transfection and subcloning of *P*. *mediterraneus* trophozoites expressing GFP

**Fig 3:**
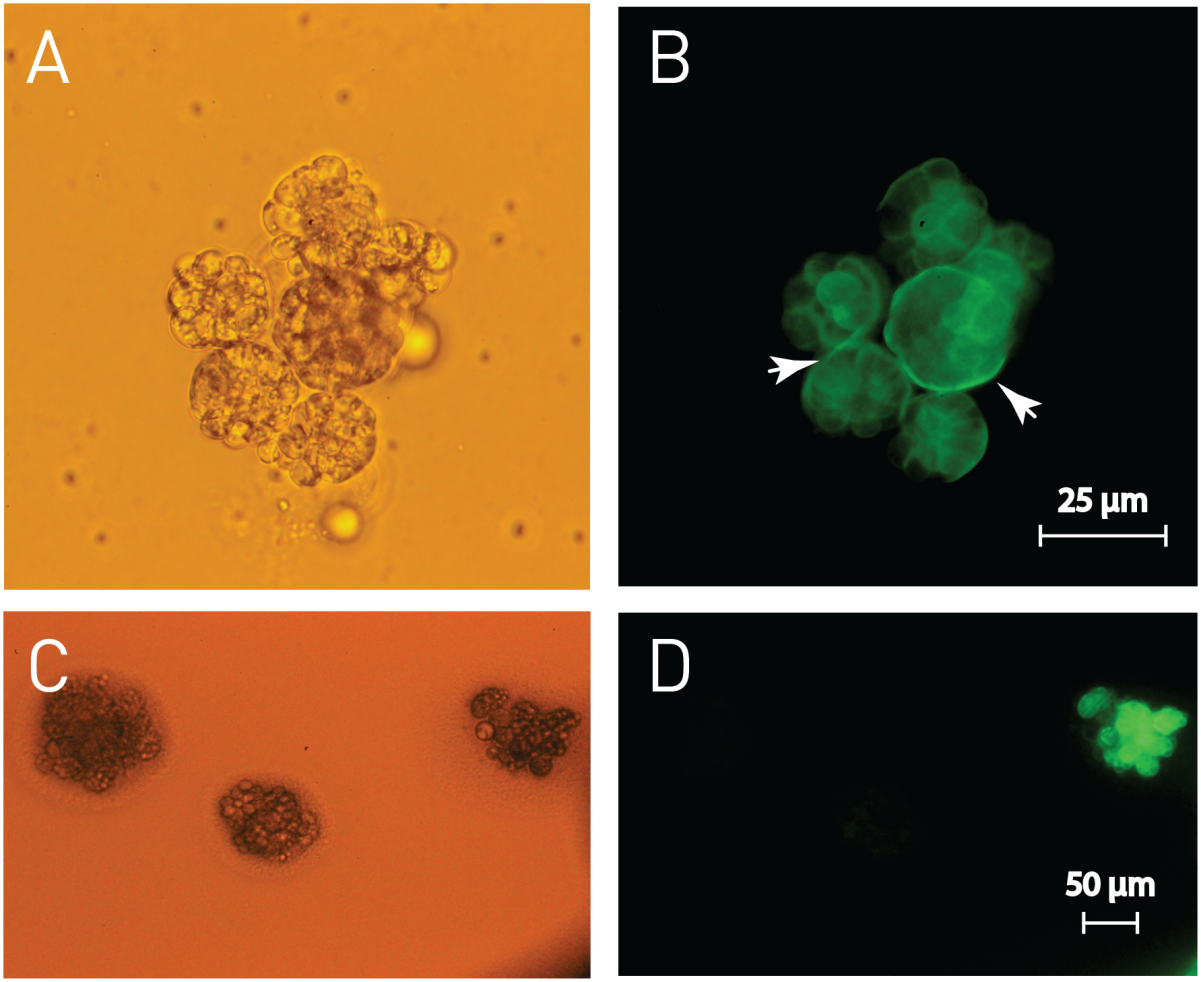
*Perkinsus mediterraneus* MOE[MOE]: GFP cloning on agar plates. **A**. *Perkinsus mediterraneus* MOE[MOE]: GFP growing in clumps in liquid medium (bright field) **B**. Blue light excitation; note the mother cell cell-wall (arrowheads). **C**. Detail of *P*. *mediterraneus* MOE[MOE]: GFP and non-fluorescent *P*. *mediterraneus* growing as single colonies after spreading the culture on the plate (bright field). **D**. Blue light excitation.

*Perkinsus marinus* transfection was developed almost one decade ago based on the highly expressed gene MOE; the same construct was used to successful transfect *P*. *olseni* [12]. Here we used the same approach to transfect *P*. *mediterraneus*, a *Perkinsus* sp. that was propagated and described from a flat oyster, *Ostrea edulis*, of Menorca, Spain. *Perkinsus mediterraneus* is characterized by growing in large clumps and for reaching lower densities in suspension cultures than other *Perkinsus* spp. [25]. The transfection vector *p*PmMOE-GFP-11, containing the flanking regions of *P*. *marinus* MOE, was able to drive transcription in *P*. *mediterraneus*. The engineered *P*. *mediterraneus* MOE[MOE]: GFP also grows in clumps as the wild type [25] with fluorescence concentrated in the outermost part of the cell, a phenotype also described for *P*. *marinus* MOE[MOE]: GFP [12] (Fig 3A and 3B). Establishing *Perkinsus* spp. cultures from the host is usually followed by subcloning by limiting dilution [3, 30] or by picking individual fluorescent trophozoites (*e*.*g*. GFP) under the microscope using a micropipette [12]. Filtered spent medium from actively growing cultures is typically added to accelerate the division of individual subcloned cells, yet cells subcloned using either of these approaches do not always proliferate [3]. Employing solid media plates we were able to grow individual *P*. *mediterraneus* colonies from low-density inocula without the addition of spent medium from actively growing cultures. We used subcloning by picking individual fluorescent trophozoites to select *P*. *mediterraneus* transfectants expressing GFP in the absence of a positive drug selection marker (*e*.*g*. selection using resistance to chloramphenicol); consequently, we tested if we would be able to see the fluorescence of plated *P*. *mediterraneus* MOE[MOE]: GFP [12] as a way to identify and select transfectants expressing GFP. Indeed, *P*. *mediterraneus* MOE[MOE]: GFP was easily visible on the plates indicating that plating can be used for identifying cells expressing tagged genes with fluorescence. Additionally, in the case of selection in liquid medium, fluorescent cells would be more difficult to see when at lower densities than non-transfected cells. Moreover, unlike in liquid medium, cells are immobilized on the surface of the solid medium allowing monitoring individual colonies over time. Selected fluorescent colonies were picked and streaked again onto fresh media plates for a second round of subcloning (Fig 3C and 3D). Subcloned colonies were further picked and successfully employed for establishing liquid cultures for expansion and cryopreservation.

### Chemosensitivity testing

**Fig 4:**
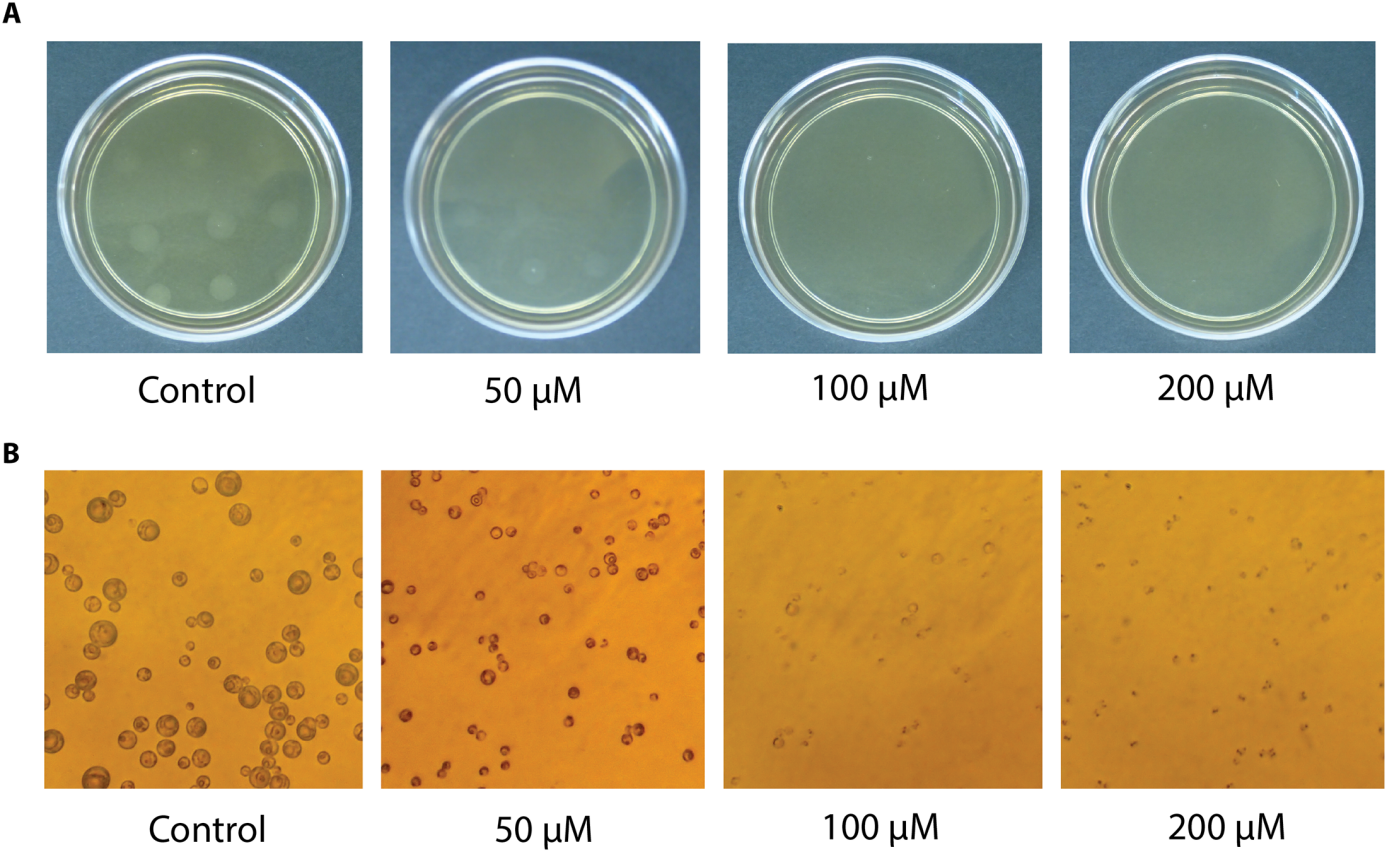
Effect of triclosan on plated *Perkinsus marinus* five days after exposure. **A.** Overview of the plates. **B.** Detail of the cells on the plates (40x).

Over the past few years the library of compounds and drugs active against *Perkinsus* spp. (tested in liquid medium cultures) has been significantly expanded [28, 31]. In particular, triclosan has been shown to inhibit *P*. *marinus* growth in liquid media with IC_50_ between 20 and 94 μM [27, 32]. To test the effect of triclosan in *P*. *marinus* growing in solid medium we choose two concentrations above and one below the IC_50_ determined for the *P*. *marinus* strain used in this study [27]. Here, we demonstrated that triclosan was also active against *P*. *marinus* in solid medium (Fig 4A) as indicated by the observation of disrupted cells and the limited formation of colonies at 50 μM or the complete absence at higher triclosan concentrations (Fig 4B). This method represents an alternative to the media-dilution method for the screening of compounds against *P*. *marinus* with the caveat that plating requires more time both in preparation and in determining the effect of compounds.

Currently, selection of transfectants of *Perkinsus* spp. relays on tagging genes with fluorescent tags (*e*.*g*. GFP) and subcloning the transfectants by selecting fluorescent cells by limiting dilution or manually pipetting individual fluorescent cells [12]. As resistance cassettes are currently being developed in several laboratories (R.F. Waller, personal communication), we foresee that infused agar plates (*e*.*g*. tetracycline, chloramphenicol) [27] will facilitate selecting transfectants with specific resistance cassettes incorporated into transfection vectors [12]. Cells carrying the plasmid would be the only ones growing on the plates, hence eliminating the need for fluorescent tagging of the genes of interest and facilitating the cloning of the transfectants.

### Potential uses of the plating technique

**Fig 5:**
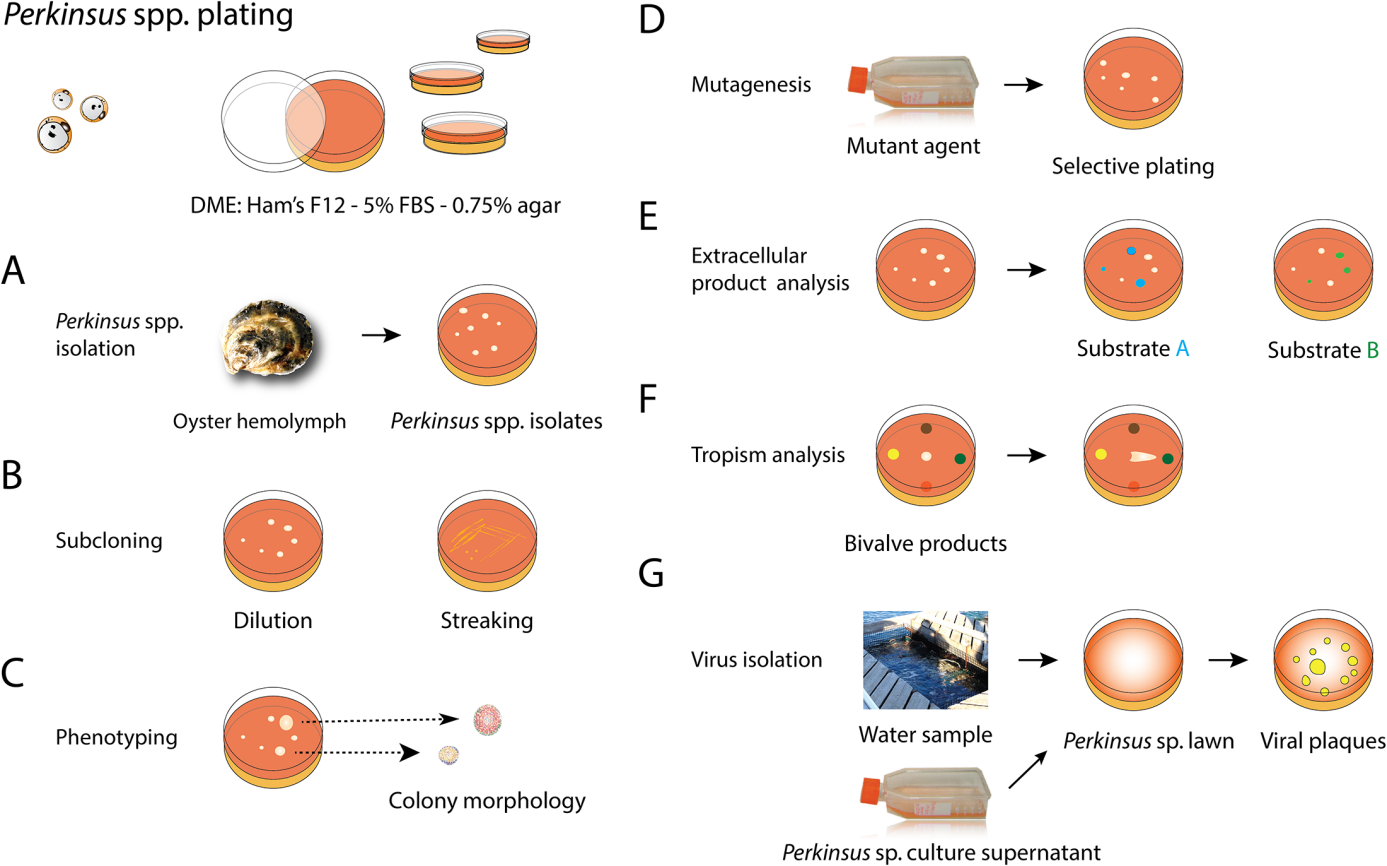
Possible applications of plating *Perkinsus marinus*. **A.** *Perkinsus* sp. Isolation. Tissue samples (*e*.*g*. hemolymph) or filtrates from waters close to bivalve aquaculture operations could be directly deposited on the plates. **B.** Subcloning of *P*. *marinus* isolates or transfectants expressing fluorescent proteins by spreading the diluted sample on the plate or by streaking (this study). **C.** Phenotyping *Perkinsus* spp. and strains based on the colony morphology. **D.** Mutagenesis. *Perkinsus* sp. culture is exposed to mutant agents and plates infused with specific inhibitor/substrates for selection depending on the nature of the mutant phenotype of interest or based on cell/colony morphology. **E.** Extracellular product analysis. The solid medium can be infused with specific substrates to analyze and compare their degradation as the *Perkinsus* sp. colony grows over time. **F.** Tropism analysis by depositing a *Perkinsus* sp. on the center of the plate and components of the bivalve host in different parts of the plate. **G.** *Perkinsus* sp. virus isolation. *Perkinsus* sp. culture supernatant or filtrates from waters close to bivalve aquaculture areas could be directly deposited on *Perkinsus* sp. lawn plates, incubate, and monitor the plates for formation of lysis plaques.

*Perkinsus marinus* is by far the marine protozoan parasite of mollusks for which more scientific resources and tools are available [6]. Here, we have added a new method for plating several *Perkinsus* spp. on a solid medium. This new technique opens the door to multiple potential applications. A few application examples, some proven in this study and other which we hypothesize are possible, are indicated in Fig 5.

Plating has been used to isolate *Trypanosoma cruzi* from the intestinal content of the kissing bug [19] and for subcloning isolates. Similarly, plating hemolymph or environmental water samples to solid media should be a straightforward method for establishing *Perkinsus* sp. isolate cultures (Fig 5A). Using this technique we have been able to recover *P*. *marinus* MOE[MOE]: GFP [12] from the hemolymph of oysters experimentally challenged with the protozoan parasite (results not shown). The presence of “races” and genetic strains of *P*. *marinus* along the coasts of the USA has been assessed on the basis of cell enlargement in RFTM [33], genetic characterization [34, 35], and sensitivity to drugs [28, 36]. The plating technique could be used for examining natural population diversity with respect to, for example, size and morphology of cells and colonies that develop on solid media from a mixed natural sample when grown on plain media or media induced with different compounds. Additionally, our method can be applicable for phenotypic (*e*.*g*. morphology and growth) characterization of *Perkinsus* spp. isolates after mutagenic treatment, an approach that has been used on other plated organisms such as yeast and algae [37–40] (Fig 5C and 5D).

Supernatant media from *Perkinsus* sp. suspension cultures contains numerous extracellular products (ECP) with enzymatic activity (*e*.*g*. proteases, glycoxydases, lipases, superoxide dismutase), that may break down host tissues into transportable components [25, 41, 42], to protect the parasite against the host immune response [43–45], and to affect the bivalve defense parameters [46]. Activity gels or assays with purified proteins have been used to demonstrate activities in ECPs and protective enzymes [25, 42, 47, 48]. Plating *Perkinsus* spp. offers an alternative method not only for detecting ECP activities by adding substrates of interest to the solid medium (*e*.*g*. plates containing gelatin for protease activity) but also for quantifying the degradation of the substrate around the colonies; similarly, by infusing the solid medium with host substrates this approach can be used for selecting clones displaying a specific trait (*e*.*g*. resistance to host defense molecules) or for comparing strain phenotypes (Fig 5E).

*Perkinsus* spp. trophozoites lack mechanical structures for active motility (*e*.*g*. gliding) [29]. However, *Perkinsus* spp. can respond to environmental and host’s cues and *Perkinsus olseni* (= *atlanticus*) appears to accumulate in the gills, an area where aggregates of *P*. *olseni* are often seen in heavily infected specimens [49, 50]. Plating offers an opportunity to study the behavior of the trophozoites on a solid medium in response to a host’s cues infused in or deposited on the agar plates (Fig 5F).

Another interesting aspect of several *Perkinsus* spp. is that they appear to be susceptible to viral infection, as shown in several ultrastructural studies that revealed virus-like particles (VLP) within trophozoites of *Perkinsus* spp. [26, 51–53]. However, these observations have been sporadic and, to the best of our knowledge, no further attempt to isolate or characterize those viruses and the consequences of infection on the protozoan host has been reported. Research on other parasitic protozoa (including some with human hosts) has revealed many interesting biological phenomena [54–56] that suggest that there is every reason to expect that viruses have a profound effect on the propagation, life style, and virulence of *Perkinsus* spp. Being able to grow *Perkinsus* spp. cells in solid media may facilitate confirming the presence and isolation of *Perkinsus* spp. viruses by plaque assays as routinely done for isolation of, for example, bacteriophages and viruses of a wide range of photosynthetic and non-photosynthetic protists [57, 58]. Plating *P*. *marinus* at high densities results in a *P*. *marinus* lawn, a necessary condition for performing plaque assays, which can be inoculated with virus-containing filtrates (<0.2 μm, to remove cellular components) from environmental seawater samples from areas with a heavy presence of *Perkinsus* spp. [59], from bivalve extracts, or from *Perkinsus* spp. cultures (Fig 5G).

In summary, we have added a new technique to the genus *Perkinsus* toolbox. This technique would bypass some methodological limitations in current studies and it has the potential for exploring new avenues to study the parasite’s biology.

## References

[1] La Peyre JF, Faisal M and Burreson EM (1993) *In vitro* propagation of the protozoan *Perkinsus marinus*, a pathogen of the eastern oyster, *Crassostrea virginica*. J Eukaryot Microbiol 40: 304–310.

[2] Kleinschuster SJ and Swink SL (1993) A simple method for the *in vitro* culture of *Perkinsus marinus*. Nautilus 107: 76–78.

[3] Gauthier JD and Vasta GR (1993) Continuous *in vitro* culture of the eastern oyster parasite *Perkinsus marinus*. J Invertebr Pathol 62: 321–323.

[4] Blackbourn J, Bower SM and Meyer GR (1998) *Perkinsus qugwadi* sp. Nov. (*incertae sedis*), a pathogenic protozoan parasite of the Japanese scallops *Patinopecten yessoensis*, cultured in British Columbia, Canada. Can J Zool 76: 942–953.

[5] http://www.atcc.org/.

[6] Fernández Robledo JA, Vasta GR and Record NR (2014) Protozoan parasites of bivalve molluscs: Literature follows culture. PLoS One 9: e100872 10.1371/journal.pone.0100872 24955977 PMC4067406

[7] http://www.ncbi.nlm.nih.gov/genome/?term=Perkinsus marinus.

[8] Joseph SJ, Fernández Robledo JA, Gardner MJ, El-Sayed NM, Kuo CH, Schott EJ, et al (2010) The Alveolate *Perkinsus marinus*: Biological insights from EST gene discovery. BMC Genomics 11: 228 10.1186/1471-2164-11-228 20374649 PMC2868825

[9] Ascenso RM (2011) Bioinformatics tools help molecular characterization of *Perkinsus olseni* differentially expressed genes. J Integr Bioinform 8: 179 10.2390/biecoll-jib-2011-179 21926442

[10] Fernández-Boo S, Villalba A and Cao A (2015) Cell proteome variability of protistan mollusc parasite *Perkinsus olseni* among regions of the Spanish coast. Dis Aquat Organ 113: 245–256. 10.3354/dao02835 25850402

[11] Fernández-Boo S, Chicano-Galvez E, Alhama J, Barea JL, Villalba A and Cao A (2014) Comparison of protein expression profiles between three *Perkinsus* spp., protozoan parasites of molluscs, through 2D electrophoresis and mass spectrometry. J Invertebr Pathol 118: 47–58. 10.1016/j.jip.2014.02.011 24607654

[12] Fernández Robledo JA, Lin Z and Vasta GR (2008) Transfection of the protozoan parasite *Perkinsus marinus*. Mol Biochem Parasitol 157: 44–53. 17996961 10.1016/j.molbiopara.2007.09.007

[13] Fernández Robledo JA, Schott EJ and Vasta GR (2008) *Perkinsus marinus* superoxide dismutase 2 (PmSOD2) localizes to single-membrane subcellular compartments. Biochem Biophys Res Commun 375: 215–219. 10.1016/j.bbrc.2008.07.162 18706398

[14] Fernández Robledo JA and Vasta GR (2010) Production of recombinant proteins from protozoan parasites. Trends Parasitol 26: 244–254. 10.1016/j.pt.2010.02.004 20189877 PMC2862126

[15] Wijayalath W, Majji S, Kleschenko Y, Pow-Sang L, Brumeanu TD, Villasante EF, et al (2014) Humanized HLA-DR4 mice fed with the protozoan pathogen of oysters *Perkinsus marinus* (Dermo) do not develop noticeable pathology but elicit systemic immunity. PLoS One 9: e87435 10.1371/journal.pone.0087435 24498105 PMC3909113

[16] Zhang H, Dungan CF and Lin S (2011) Introns, alternative splicing, spliced leader trans-splicing and differential expression of pcna and cyclin in *Perkinsus marinus*. Protist 162: 154–167. 10.1016/j.protis.2010.03.003 20650682

[17] Lin Z, Fernández Robledo JA, Cellier MF and Vasta GR (2011) The natural resistance-associated macrophage protein from the protozoan parasite *Perkinsus marinus* mediates iron uptake. Biochemistry 50: 6340–6355. 10.1021/bi200343h 21661746

[18] Carruthers VB and Cross GA (1992) High-efficiency clonal growth of bloodstream- and insect-form *Trypanosoma brucei* on agarose plates. Proc Natl Acad Sci USA 89: 8818–8821. 1528898 10.1073/pnas.89.18.8818 PMC50012

[19] Mondragon A, Wilkinson SR, Taylor MC and Kelly JM (1999) Optimization of conditions for growth of wild-type and genetically transformed *Trypanosoma cruzi* on agarose plates. Parasitology 118 (Pt 5): 461–467. 10363279 10.1017/s0031182099004230

[20] Goldberg SS and Chiari E (1980) Growth and isolation of single colonies of *Trypanosoma cruzi* on solid medium. The Journal of Parasitology 66: 677–679. 6999145

[21] Muniaraj M, Sinha PK and Das P (2010) Antileishmanial activity of drug infused mini-agar plates on *Leishmania donovani* promastigotes. Trop Biomed 27: 657–661. 21399608

[22] Vassella E and Boshart M (1996) High molecular mass agarose matrix supports growth of bloodstream forms of pleomorphic *Trypanosoma brucei* strains in axenic culture. Mol Biochem Parasitol 82: 91–105. 8943153 10.1016/0166-6851(96)02727-2

[23] Lee MG and Van der Ploeg LH (1989) Colonies of procyclic *Trypanosoma brucei* on semi-solid agarose plates. Mol Biochem Parasitol 34: 193–196. 2710171 10.1016/0166-6851(89)90011-x

[24] Brun R and Schonenberger (1979) Cultivation and *in vitro* cloning or procyclic culture forms of *Trypanosoma brucei* in a semi-defined medium. Short communication. Acta Trop 36: 289–292. 43092

[25] Casas SM, Reece KS, Li Y, Moss JA, Villalba A and La Peyre JF (2008) Continuous culture of *Perkinsus mediterraneus*, a parasite of the European flat oyster *Ostrea edulis*, and characterization of its morphology, propagation, and extracellular proteins *in vitro*. J Eukaryot Microbiol 55: 34–43. 10.1111/j.1550-7408.2008.00301.x 18251801

[26] Gauthier JD, Vasta G. R. (1995) *In vitro* culture of the Eastern parasite *Perkinsus marinus*: optimization of the methodology. J Invertebr Pathol 66: 156–168.

[27] Shridhar S, Hassan K, Sullivan DJ, Vasta GR and Fernández Robledo JA (2013) Quantitative assessment of the proliferation of the protozoan parasite *Perkinsus marinus* using a bioluminescence assay for ATP content. Int J Parsitol: Drug Drug Resist 3: 85–92. 10.1016/j.ijpddr.2013.03.001 PMC3862420 24533297

[28] Alemán Resto Y and Fernández Robledo JA (2014) Identification of MMV Malaria Box inhibitors of *Perkinsus marinus* using an ATP-based bioluminescence assay. PLoS One 9: e111051 10.1371/journal.pone.0111051 25337810 PMC4206467

[29] Perkins FO (1996) The structure of *Perkinsus marinus* (Mackin, Owen and Collier, 1950) Levine, 1978 with comments on taxonomy and phylogeny of *Perkinsus* spp. J Shellfish Res 15: 67–87.

[30] Robledo JA, Nunes PA, Cancela ML and Vasta GR (2002) Development of an *in vitro* clonal culture and characterization of the rRNA gene cluster of *Perkinsus atlanticus*, a protistan parasite of the clam *Tapes decussatus*. J Eukaryot Microbiol 49: 414–422. 12425530 10.1111/j.1550-7408.2002.tb00221.x

[31] Leite MA, Alfonso R and Cancela ML (2011) Herbicides and Protozoan Parasite Growth Control: Implications for New Drug Development In: Larramendy M., Soloneski S., editor editors. Herbicides, Theory and Applications. Rijeka, Croatia: InTech pp. 567–580.

[32] Lund ED, Soudant P, Chu FL, Harvey E, Bolton S and Flowers A (2005) Effects of triclosan on growth, viability and fatty acid synthesis of the oyster protozoan parasite *Perkinsus marinus*. Dis Aquat Organ 67: 217–224. 16408837 10.3354/dao067217

[33] Bushek D, Ford S. E. Allen S. K. Jr (1994) Evaluation of methods using Ray's fluid thioglycollate medium for diagnosis of *Perkinsus marinus* infection ub the Eastern oyster *Crassostrea virginica*. Ann Rev Fish Dis 4: 201–217.

[34] Reece K, Bushek D, Hudson K and Graves J (2001) Geographic distribution of *Perkinsus marinus* genetic strains along the Atlantic and Gulf coasts of the USA. Mar Biol 139: 1047–1055.

[35] Robledo JAF, Wright AC, Marsh AG and Vasta GR (1999) Nucleotide sequence variability in the nontranscribed spacer of the rRNA locus in the oyster parasite *Perkinsus marinus*. J Parasitol 85: 650–656. 10461944

[36] Panko C, Volety A, Encomio V and Barreto J (2006) Evaluation of the antimalarial drug quinine as a potential chemotherapeutic agent for the eastern oyster parasite, *Perkinsus marinus*. J Shellfish Res 25: 760.

[37] Gil de Prado E, Rivas EM, de Siloniz MI, Diezma B, Barreiro P and Peinado JM (2014) Quantitative analysis of morphological changes in yeast colonies growing on solid medium: the eccentricity and Fourier indices. Yeast 31: 431–440. 10.1002/yea.3036 25100432

[38] Chen L, Noorbakhsh J, Adams RM, Samaniego-Evans J, Agollah G, Nevozhay D, et al (2014) Two-dimensionality of yeast colony expansion accompanied by pattern formation. PLoS Comput Biol 10: e1003979 10.1371/journal.pcbi.1003979 25504059 PMC4263361

[39] Barberio C, Bianchi L, Pinzauti F, Lodi T, Ferrero I, Polsinelli M, et al (2007) Induction and characterization of morphologic mutants in a natural *Saccharomyces cerevisiae* strain. Can J Microbiol 53: 223–230. 17496970 10.1139/W06-132

[40] Deschamps P, Guillebeault D, Devassine J, Dauvillee D, Haebel S, Steup M, et al (2008) The heterotrophic dinoflagellate *Crypthecodinium cohnii* defines a model genetic system to investigate cytoplasmic starch synthesis. Eukaryot Cell 7: 872–880. 10.1128/EC.00461-07 18310353 PMC2394971

[41] Earnhart CG, Vogelbein MA, Brown GD, Reece KS and Kaattari SL (2004) Supplementation of *Perkinsus marinus* cultures with host plasma or tissue homogenate enhances their infectivity. Appl Environ Microbiol 70: 421–431. 14711671 10.1128/AEM.70.1.421-431.2004 PMC321304

[42] McLaughlin SM, Elsayed EE and Faisal M (2000) Analysis of extracellular proteins of two *Perkinsus* spp. isolated from the softshell clam *Mya arenaria in vitro*. Comp Biochem Physiol B Biochem Mol Biol 126: 587–598. 11026671 10.1016/s0305-0491(00)00231-5

[43] Schott EJ, Pecher WT, Okafor F and Vasta GR (2003) The protistan parasite *Perkinsus marinus* is resistant to selected reactive oxygen species. Exp Parasitol 105: 232–240. 14990317 10.1016/j.exppara.2003.12.012

[44] Schott EJ, Robledo JA, Wright AC, Silva AM and Vasta GR (2003) Gene organization and homology modeling of two iron superoxide dismutases of the early branching protist *Perkinsus marinus*. Gene 309: 1–9. 12727353 10.1016/s0378-1119(03)00469-4

[45] Schott EJ and Vasta GR (2003) The PmSOD1 gene of the protistan parasite *Perkinsus marinus* complements the sod2Delta mutant of *Saccharomyces cerevisiae*, and directs an iron superoxide dismutase to mitochondria. Mol Biochem Parasitol 126: 81–92. 12554087 10.1016/s0166-6851(02)00271-2

[46] Garreis KA, La Peyre JF and Faisal M (1996) The effects of *Perkinsus marinus* extracellular products and purified proteases on oyster defence parameters *in vitro*. Fish Shellfish Immunol 6: 581–597.

[47] Ahmed H, Schott EJ, Gauthier JD and Vasta GR (2003) Superoxide dismutases from the oyster parasite *Perkinsus marinus*: purification, biochemical characterization, and development of a plate microassay for activity. Anal Biochem 318: 132–141. 12782041 10.1016/s0003-2697(03)00192-1

[48] Asojo OA, Schott EJ, Vasta GR and Silva AM (2006) Structures of PmSOD1 and PmSOD2, two superoxide dismutases from the protozoan parasite *Perkinsus marinus*. Acta Crystallograph Sect F Struct Biol Cryst Commun 62: 1072–1075. 10.1107/S1744309106040425 PMC2225229 17077482

[49] Azevedo C (1989) Fine structure of *Perkinsus atlanticus* n. sp. (Apicomplexa, Perkinsea) parasite of the clam *Ruditapes decussatus* from Portugal. J Parasitol 75: 627–635. 2760774

[50] Montes JF, Del Rio JA, Durfort M and Garcia-Valero J (1997) The protozoan parasite *Perkinsus atlanticus* elicits a unique defensive response in the clam *Tapes semidecussatus*. Parasitology 114: 339–350.

[51] Azevedo C (1990) Virus-like particles in *Perkinsus atlanticus* (Apicomplexa, Perkinsidae). Dis Aquat Organ 9: 63–65.

[52] Coss CA, Robledo JAF, Ruiz GM and Vasta GR (2001) Description of *Perkinsus andrewsi* n. sp. isolated from the Baltic clam (*Macoma balthica*) by characterization of the ribosomal RNA locus, and development of a species-specific PCR-based diagnostic assay. J Eukaryot Microbiol 48: 52–61. 11249193 10.1111/j.1550-7408.2001.tb00415.x

[53] Coss CA, Robledo JAF and Vasta GR (2001) Fine structure of clonally propagated *in vitro* life stages of a *Perkinsus* sp. isolated from the Baltic clam *Macoma balthica*. J Eukaryot Microbiol 48: 38–51. 11249192 10.1111/j.1550-7408.2001.tb00414.x

[54] Miles MA (1988) Viruses of parasitic protozoa. Parasitol Today 4: 289–290. 15463003 10.1016/0169-4758(88)90023-3

[55] Wang AL and Wang CC (1991) Viruses of parasitic protozoa. Parasitol Today 7: 76–80. 15463448 10.1016/0169-4758(91)90198-w

[56] Raoult D, Audic S, Robert C, Abergel C, Renesto P, Ogata H, et al (2004) The 1.2-megabase genome sequence of Mimivirus. Science 306: 1344–1350. 15486256 10.1126/science.1101485

[57] Middelboe M, Chan AM and Bertelsen SK (2010) Isolation and life cycle characterization of lytic viruses infecting heterotrophic bacteria and cyanobacteria In: W M. G.. Wilhelm S. W., and Suttle C. A., editor editors. Manual of Aquatic Viral Ecology.ASLO pp. 118–133.

[58] Nagasaki K and Bratbak G (2010) Isolation of viruses infecting photosynthetic and nonphotosynthetic protists In: W M. G.. Wilhelm S. W., and Suttle C. A., editor editors. Manual of Aquatic Viral Ecology. ASLO pp. 92–101.

[59] Marquis ND, Record NR and Fernández Robledo JA (2015) Survey for protozoan parasites in Eastern oysters (*Crassostrea virginica*) from the Gulf of Maine using PCR-based assays. Parasitol Int 64: 299–302. 10.1016/j.parint.2015.04.001 25889457

